# A Robust Faster R-CNN Model with Feature Enhancement for Rust Detection of Transmission Line Fitting

**DOI:** 10.3390/s22207961

**Published:** 2022-10-19

**Authors:** Zhimin Guo, Yangyang Tian, Wandeng Mao

**Affiliations:** Electric Power Research Institute, State Grid Henan Electric Power Company, Zhengzhou 450007, China

**Keywords:** rust detection, transmission lines fitting, object recognition, faster R-CNN, transmission safety

## Abstract

Rust of transmission line fittings is a major hidden risk to transmission safety. Since the fittings located at high altitude are inconvenient to detect and maintain, machine vision techniques have been introduced to realize the intelligent rust detection with the help of unmanned aerial vehicles (UAV). Due to the small size of fittings and disturbance of complex environmental background, however, there are often cases of missing detection and false detection. To improve the detection reliability and robustness, this paper proposes a new robust Faster R-CNN model with feature enhancement mechanism for the rust detection of transmission line fitting. Different from current methods that improve feature representation in front end, this paper adopts an idea of back-end feature enhancement. First, the residual network ResNet-101 is introduced as the backbone network to extract rich discriminative information from the UAV images. Second, a new feature enhancement mechanism is added after the region of interest (ROI) pooling layer. Through calculating the similarity between each region proposal and the others, the feature weights of the region proposals containing target object can be enhanced via the overlaying of the object’s representation. The weight of the disturbance terms can then be relatively reduced. Empirical evaluation is conducted on some real-world UAV monitoring images. The comparative results demonstrate the effectiveness of the proposed model in terms of detection precision and recall rate, with the average precision of rust detection 97.07%, indicating that the proposed method can provide an reliable and robust solution for the rust detection.

## 1. Introduction

Due to long-term exposure to the wild environment, transmission line fittings are prone to defects such as aging, damage and rust, resulting in heavy risk to the transmission safety. It is significantly important to detect and deal with the rust of transmission line fitting in a timely manner. Presently, unmanned aerial vehicle (UAV) inspection has replaced labor routing inspection in many scenarios due to some merits such as no terrain limitation, fast speed, high efficiency, low labor costs, strong safety and so on. In the UAV inspection mode, however, UAVs generally collect monitoring data for the artificial check, which is with low efficiency. Machine vision with artificial intelligence techniques is currently becoming a promising tool to analyze the UAV monitoring data, and has shown prevailing performance compared artificial check. It is of great significance to develop a robust and accurate rust detection method for transmission line fittings.

From the theoretical perspective of machine vision, the rust detection problem can be viewed as the problem of object detection [1]. With the rapid development of convolutional neural network (CNN), deep learning techniques have become a promising tool in object detection [2]. In summary, these techniques can be divided into two strategies: one-stage detection and two-stage detection. The one-stage algorithm, such as YOLO [3], SSD [4,5], RetinaNet [6], uses a unified deep neural network (e.g., CNN) for feature extraction, target classification and bounding box regression, achieving end-to-end object detection. It has a faster detection speed and relatively lower detection accuracy. The two-stage algorithms, mainly the variants of R-CNN, i.e., R-CNN [7], Fast R-CNN [8], Faster R-CNN [9] and Mask R-CNN [10], adopt a classical sliding window mechanism to extract interested region and then carry out classification with the features of the regions. In these algorithms, Faster R-CNN is on par with, or even outperforms, the other algorithms in terms of detection accuracy. Nevertheless, the classical Faster R-CNN still has some limitations in the detection of small-size objects, especially under complex background. Many studies have been devoted to overcoming the limitations. For instance, Cui et al. [11] adopted a feature pyramid network in Faster R-CNN with attention module. By highlighting the saliency of object’s features, the detection accuracy can be improved. Lim et al. [12] introduced a residual attention mechanism to obtain rich information of small-size objects. Aside from considering feature representation, Xue et al. [13] also introduced coordinate attention mechanism into Faster R-CNN for incorporating the location information that is believed helpful to the channel information. Hong et al. [14] designed a quartile attention mechanism that uses four branches to capture internal and cross-latitude interactions between channels and spatial locations, making better use of contextual information.

**Figure 1:**
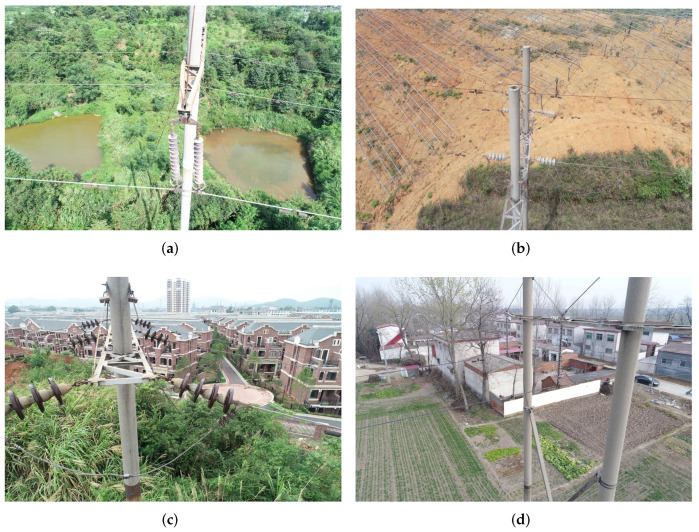
Rust detection examples of transmission line fitting with different challenges: (**a**,**b**) are of small object, while (**c**,**d**) are with complex background.

**Figure 2:**
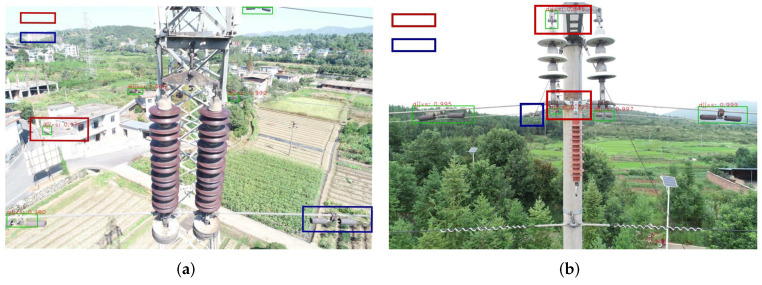
Examples of missing detection and false detection using Lim’s method [12] that is an improved version of Faster R-CNN for small-size object detection, where (**a**,**b**) are the two examples regarding of missing detection and false detection.

These studies can improve the detection robustness under challenging environments by extracting rich semantic information. According to our empirical study, however, these methods do not work well in the rust detection of transmission line fittings. The reason is that the rust detection of transmission line fittings has some special challenges. In most actual applications, the transmission line is long and widely distributed, leading to complex background for the detection. Too many disturbance items such as tree, car, village, house, etc., exist and raise false detection. Moreover, UAV graph usually contains several fittings, each of which has relatively small size, also raising missing detection. Figure 1 shows some real-world examples for each challenge. It is clear that the small size of fittings, as well as various kinds of disturbance items, brings heavy obstacle for the rust detection. The current methods all work to improve feature representation in front end, e.g., using attention mechanism and pyramid architecture. However, for the rust detection, these front-end improvements cannot guarantee the valid detection for small-size fittings and effectively eliminate the disturbance from the background environment. According to our literature survey, there have been some studies for solving similar problems. For instance, Zhai et al. [15] proposed a new cascade reasoning graph network for multi-fitting detection on transmission lines. This network incorporates three kinds of domain knowledge, i.e., co-occurrence knowledge, semantic knowledge and spatial knowledge, to represent the co-relation of different mini-size fittings. With these knowledge reasoned by graph attention network, more discriminative features can be extracted based on the original visual features to recognize and position the fittings. However, this method still works in front end and is devoted to feature enhancement before generating accurate proposals. It aims to develop the detection accuracy and does not consider the disturbance of complex background which will reduce the detection robustness. As shown in Figure 2, missing detection, as well as false detection, has occurred many times in our experiment when running the methods discussed above. For an actual applications, missing detection and false detection should be significantly avoided from the rust detection, especially in online scenarios. For online tour-inspection, UAVs, which are equipped the detection algorithms, need to provide more reliable and robust detection results. It is necessary to enhance the feature representation of fittings based on the current front-end techniques to improve the detection accuracy and robustness.

Based on the analysis mentioned above, the main challenge for the rust detection of fittings in complex environment is developing feature representations of the fittings against the background disturbance. We observe an interesting phenomenon from our empirical evaluations. Despite of many cases of missing detection and false detection, Faster R-CNN can still obtain the interesting regions, most of which have a certain degree of feature representation of the fittings. In other words, most of the region proposals in Faster R-CNN actually are related to the fitting object. Then it motivates us a new idea: enhance the feature representation from these regions themselves. Following this idea, we build a new Faster R-CNN model for the robust rust detection of transmission line fitting in this paper. The backbone network, VGG16 network, is replaced by a deeper network ResNet-101 for extracting more rich information about the fitting object from UAV images. More importantly, a new feature enhancement mechanism is built after the region of interest (RoI) pooling layer to improve the feature representation of the regions that have real fittings. The weight of the disturbance terms can then be relatively reduced. Comparative results on some real-world UAV monitoring images verify that the proposed model can significantly increase the detection accuracy and robustness.

The main contributions of this paper can be summarized as follows: (1) From the application perspective, this paper proposes an lightweight but effective solution for the rust detection of transmission line fittings. The proposed method is simple and of high accuracy as well as robustness. To our best knowledge, the study of the rust detection for transmission line fittings is still at its infancy. (2) From the theoretical perspective, this paper constructs a new feature enhancement mechanism in the back end of classical object detection algorithms. Different from most of current methods, this mechanism can further enhance the feature representation based on the generated features. This mechanism can apply for the current two-stage detection methods without too much modification on the algorithmic architecture. We believe this mechanism can provide a different aspect to improve the detection reliability and robustness.

The remaining part of this paper is as follows. Section 2 is dedicated to the implementation of the classical Faster R-CNN. Section 3 describes the proposed model in detail. Section 4 carries out a set of comparative experiments, followed by a conclusion in Section 5.

## 2. Background of Faster R-CNN

Faster R-CNN was developed from R-CNN and Fast R-CNN. R-CNN is the first algorithm to apply CNN to an object detection task. It uses a selective search algorithm to obtain region proposals with fine-tuning the CNN, and trains a support vector machine (SVM) classifier that also performs border regression. This method does not work end-to-end. Based on the spatial pyramid pooling network (SPP-Net [16]), Fast R-CNN inputs the whole image instead of each candidate region into R-CNN for feature extraction, also with the region proposals generated through selective search. The biggest improvement of Faster R-CNN is the use of region proposal network (RPN) to generate regions of interest (ROI), no longer using the selective search strategy again. Another interesting point is that the whole training process can run under GPU environment, indicating computationally inexpensive.

**Figure 3:**
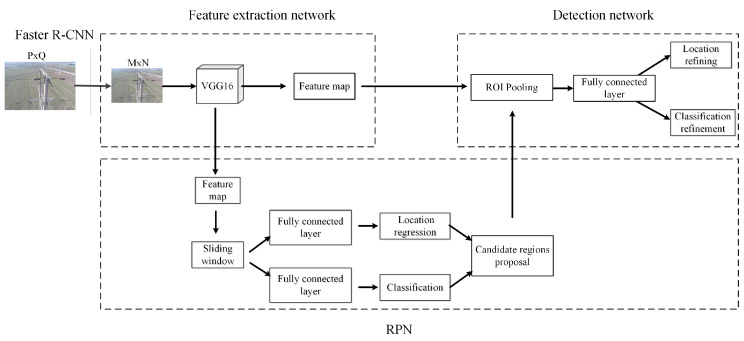
Architecture of Faster R-CNN. To improve readability, we take the rust detection problem as the background.

The classical Faster R-CNN algorithm is composed of an RPN network and a Fast R-CNN network. The whole architecture includes four parts: convolution layer, RPN layer, ROI pooling layer and classification regression layer, as shown in Figure 3. To improve readability, here we take the rust detection problem as an example to describe the algorithmic details.

Faster R-CNN first scales each UAV image of size P×Q to the size of M×N, then inputs the image to a CNN network (e.g., the commonly used VGG16) to obtain a feature map. The feature map is then fed into the RPN that generates region proposals on the feature map. The object category, as well as its position, in the region proposals can be obtained through the classification regression layer. Specifically, the RPN distinguishes between the foreground and background of region proposals, and outputs the region proposal in the foreground region. The ROI pooling layer reshape the region proposal in foreground region to a fixed size (7×7) by combining the CNN features and RPN information. The region is connected to a detection network for judging the object category and fine-tuning its position as well.

**Figure 4:**
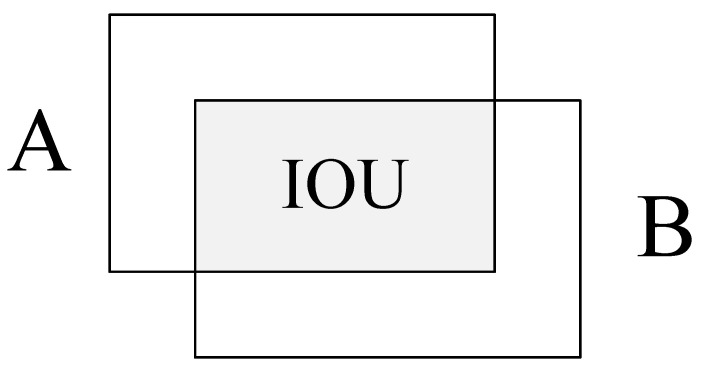
Schematic drawing of IoU calculation, where A and B are the ground-truth box and region proposal respectively.

In Figure 3, the foreground classification is the key. It requires to compare the region proposals with the ground-truth box manually annotated by experts, and further calculates the intersection ratio of the two boxes, defined as Intersection over Union (IoU): $IoU=\frac{A\cap B}{A\cup B}$, just as shown in Figure 4. When the IoU of one region proposal is greater than 0.7, the region is set as positive sample, i.e., the foreground. If the IoU < 0.3, the region is set as negative sample, i.e., the background. The region proposal with IoU value of 0.3–0.7 is not involved in the training. The positive and negative samples are then used to train RPN.

## 3. The Proposed Faster R-CNN Model with Feature Enhancement

**Figure 5:**
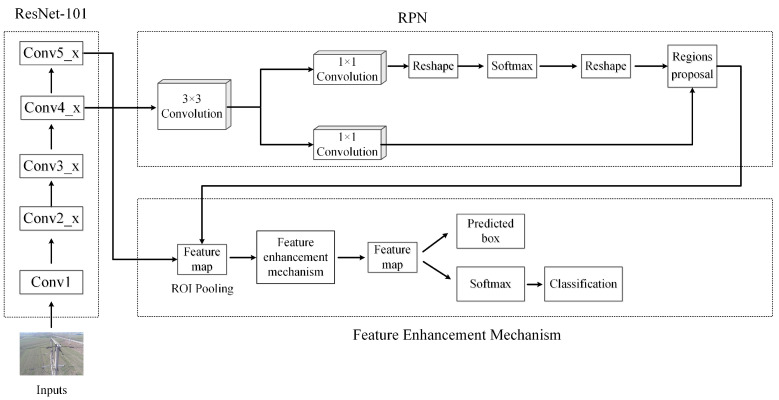
Structure of the proposed new Faster R-CNN model with feature enhancement.

In this section, a new Faster R-CNN model is proposed, in which two developments are made: updating the backbone network using the residual network ResNet-101, and designing a new feature enhancement mechanism after the ROI pooling layer. The structure of the proposed model is shown in Figure 5. The details will be elaborated as follows.

### 3.1. Feature Extraction Network with ResNet-101

**Figure 6:**
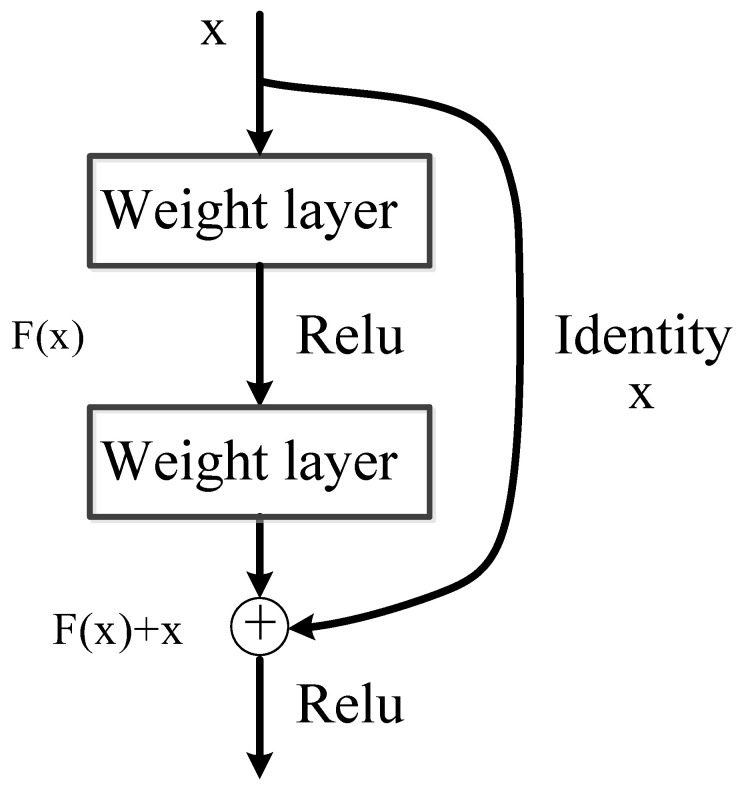
Structure of residual connection.

It has been proven that deeper network is capable of extracting more robust feature representations from images [17]. However too deep a network would raise gradient disappearance and gradient explosion. Residual connection is an effective trick to extend the depth of a deep convolutional network [18], as shown in Figure 6. Here we adopt a version of a well-known residual network, named ResNet-101 network, as the backbone network of Faster R-CNN. This network is believed to obtain richer feature information, thereby improving the feature representation for the detection. The structure is listed in Table 1. Since ResNet-101 has been widely studied, we would not analyze it in detail. Please find the reference [18] for the implementation details.

### 3.2. Feature Enhancement Mechanism

Motivated by the self-attention mechanism that can extract richer information by learning the similarity between the target object and the other ones, this section builds a new back-end feature enhancement mechanism after the ROI pooling layer rather than in the feature extraction network. Through calculating the similarity between each region proposal and the others, the feature weights of the region proposals containing target object can be enhanced via the overlaying of the object’s representation. This operation is based on the observation: the majority of the obtained region proposals contain fitting objects, and their feature representations are essentially similar. Then we can put weights on the features of the obtained region proposals. The weight on the region with fittings is pushed to be greater, while the weight on the region with disturbance item is reduced. Then the feature representation of the actual fittings can be enhanced to reach a more robust detection. This operation is called *feature enhancement mechanism*.

Specifically, denote the input as the feature map $R_{i}$ that is the output of ROI pooling layer. The calculation process is as follows:

(1) Without loss of generality, calculate the similarity between the 1st region proposal and the other ones, and get the weight $\alpha_{1,i}$:

(1) $$\alpha_{1,i}=\frac{dot(R_{1},R_{i})}{\sqrt{d_{k}}}$$

where $R_{1}$ and $R_{i}$ are the feature map of the 1st and *i*th region proposal respectively, dot(·,·) means dot product that is chosen as the similarity measure, $d_{k}$ is the input feature dimension. Certainly, different similarity measures can also be adopted.

**Figure 7:**
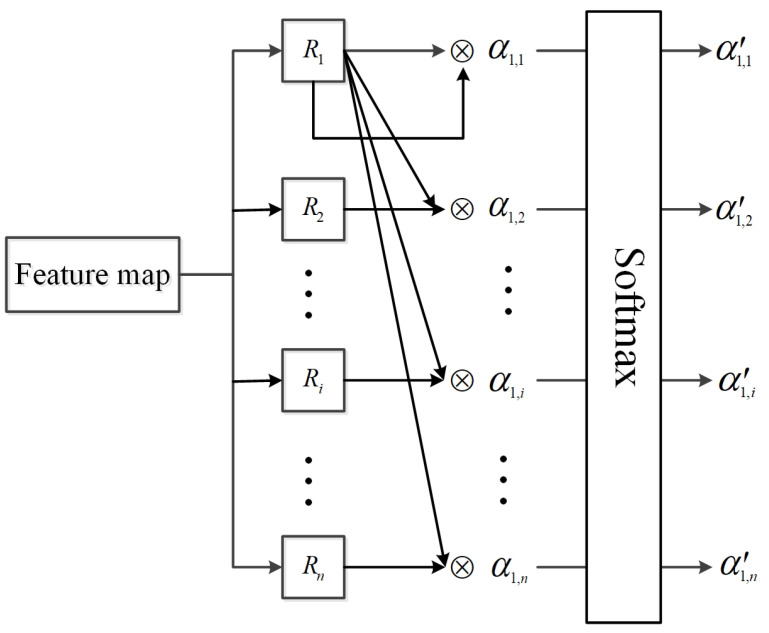
Sketch of calculating the similarity between region proposals.

(2) Normalize the weight $\alpha_{1,i}$ via the Softmax layer to obtain the final weights $\alpha_{1,i}^{\prime }$, as shown in Equation (2). This operation can also be visualized in Figure 7. Obviously, $\alpha_{1,i}^{\prime }$ indicates the influence of the i-th region proposal on the 1st region proposal.

(2) $$\alpha_{{}_{1,i}}^{\prime }=\frac{\exp(\alpha_{1,i})}{\sum_{}^{}{}_{j}\exp\left(\alpha_{1,j}\right)}$$

**Figure 8:**
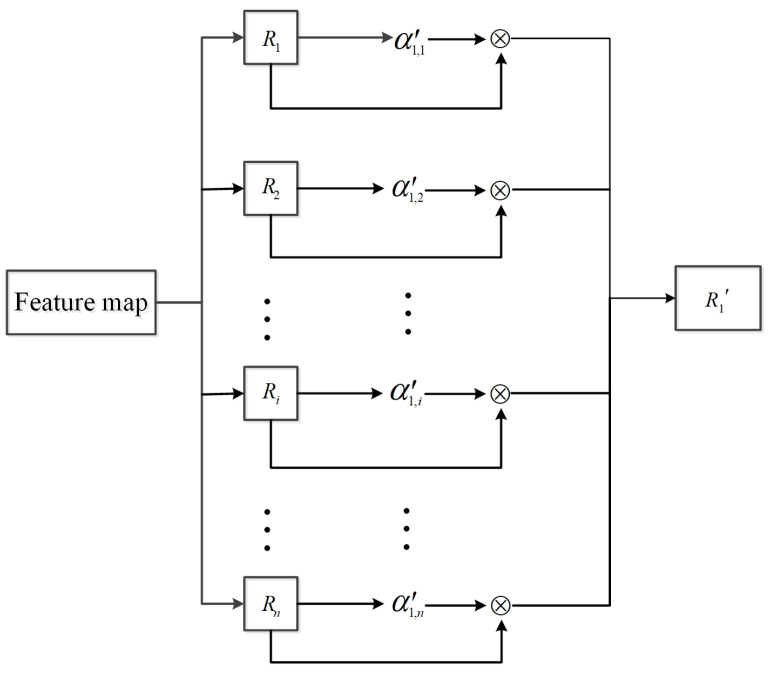
Sketch of calculating the output feature.

(3) Multiply $\alpha_{1,i}^{\prime }$ by $R_{i}$ and sum up all region proposals to obtain an output $\alpha_{1}^{\prime }$ that has the same dimension as the input data, as shown in Equation (3). The operation can be visualized in Figure 8.

(3) $$\alpha_{1}^{\prime }=\sum_{i}^{}\alpha_{1,i}^{\prime }R_{i}$$

From the analysis mentioned above, the feature enhancement mechanism can increase the weight of the target region proposals by calculating the similarity between the obtained regions, which helps to eliminate missing detection. Obviously, for the regions proposal containing disturbance item, the weight will be relatively decreased, which helps to lessen false detection.

### 3.3. Loss Function

The loss of the whole network mainly consist of classification loss $L_{cls}$ and regression loss $L_{reg}$, as follows:

(4) $$L\left(\left\{p_{i}\right\},\left\{t_{i}\right\}\right)=\frac{1}{N_{cls}}\sum_{i}L_{cls}\left(p_{i},p_{i}^{*}\right)+\lambda \frac{1}{N_{reg}}\sum_{i}p_{i}^{*}L_{reg}\left(t_{i},t_{i}^{*}\right)$$

where *i* is the anchor index, $p_{i}$ is the probability of the i-th anchor to be predicted as the ground-truth label, $p_{i}^{*}=1$ if it is a positive sample, otherwise $p_{i}^{*}=0$. $t_{i}$ is a vector representing the four parameterized coordinates of the predicted bounding box, and $t_{i}^{*}$ is that of the ground-truth box associated with a positive anchor. λ is the regularization parameter to tradeoff $L_{cls}$ and $L_{reg}$. The calculation of $L_{cls}$ and $L_{reg}$ are as follows:

(5) $$L_{cls}\left(p_{i},p_{i}^{*}\right)=-\log\left[p_{i}p_{i}^{*}+\left(1-p_{i}\right)\left(1-p_{i}^{*}\right)\right]$$

(6) $$L_{reg}\left(t_{i},t_{i}^{*}\right)=\sum_{i\in \left\{x,y,w,h\right\}}smooth_{L_{1}}\left(t_{i}-t_{i}^{*}\right)$$

where $\left\{x,y,w,h\right\}$ denotes the two coordinates of the box center, width and height, $smooth_{L_{1}}\left(x\right)=\left\{{}_{|x|-0.5\;\mathrm{otherwise}}^{0.5x^{2}\;\;\mathrm{if}|x|<1}\right.$.

**Figure 9:**
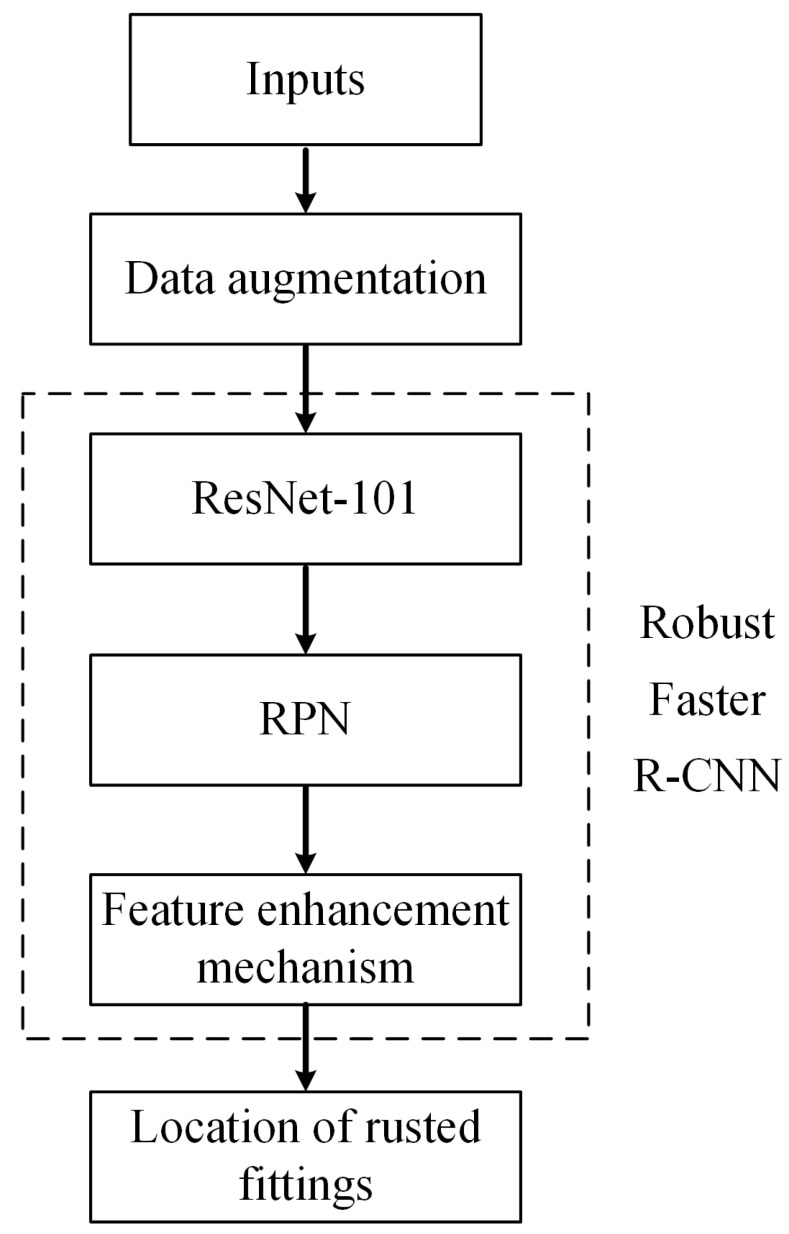
Flowchart of the whole methodology.

To improve the readability of the proposed method, we provide the flowchart of the methodology in Figure 9. The key of the methodology is the proposed feature enhancement mechanism. Please note that this mechanism can also be applied to the other two-stage object detection architectures.

## 4. Experimental Results

In this section, the effectiveness of the proposed model is verified. The programming environment is Linux Mint 19.2, PyTorch 1.0 and CUDA10.2, configured with GeForce RTX1080 graphics card.

### 4.1. Dataset Preprocessing

**Figure 10:**
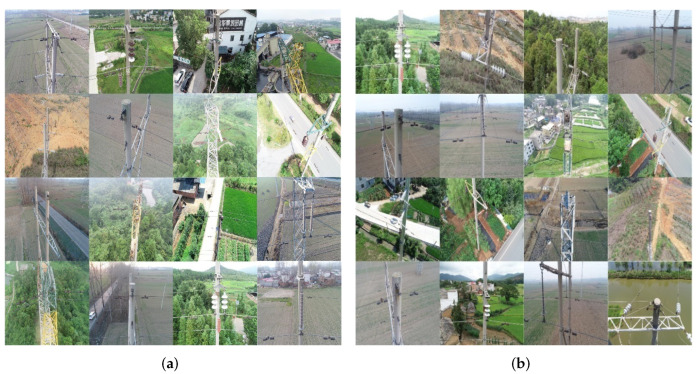
Examples of UAV images used in this experiment. For better illustrative effect, we divide the examples into the two groups, as shown in the subfigures (**a**,**b**).

**Figure 11:**
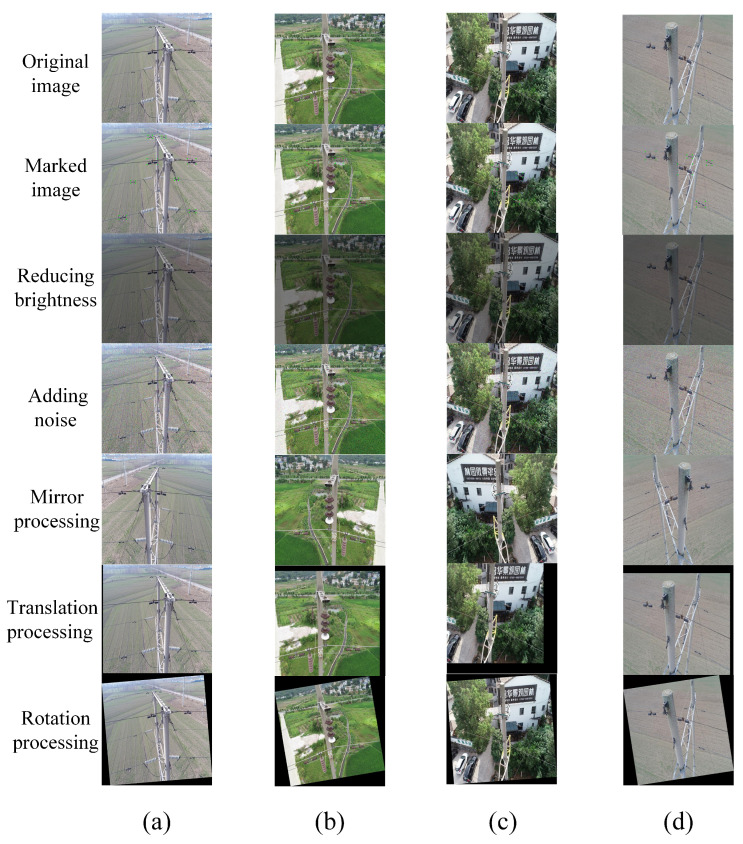
UAV images of transmission line fittings after data enhancement. The columns (**a**–**d**) are the four examples to show the effect of data enhancement..

The UAV images used in this experiment come from our real-world application, as shown in Figure 10. The dataset consists of 245 images for training and 105 images for test, which were collected from southern China. We use the LabelImg software to mark the images containing transmission line fittings as ’Fittings’. To increase the amount of training data, the images are preprocessed through data enhancement, such as adjusting brightness, adding noise, mirror processing, translation and rotation processing. The effect after data enhancement is shown in Figure 11.

### 4.2. Ablation Validation

In the proposed model, we integrate ResNet-101 and feature enhancement mechanism in Faster R-CNN. To evaluate the effect of each component, we set up two ablation experiments for the evaluation.

#### 4.2.1. Change of Backbone Network

**Figure 12:**
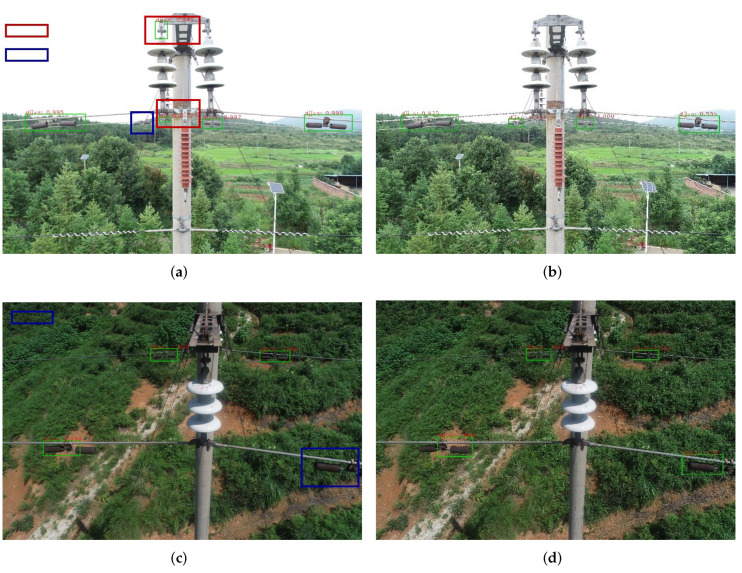
Comparative results of different backbone networks, where (**a**,**c**) are with VGG16, (**b**,**d**) are with ResNet-101.

To evaluate the effect of feature extraction network, we replace the ResNet-101 by another CNN network, i.e., VGG16. The comparative results are shown in Figure 12. It is clear that ResNet-101 can better recognize the rusted fittings with lower missing detection rate. It can demonstrate that deeper network can enhance the feature learning of the target object by extracting abundant feature information, which enables the model to obtain high accuracy in the following detection. The stronger the learning ability of the model is, the more robust the detection and the higher the detection accuracy will be. However, we also find that the phenomenon of missing detection has not been completely eliminated, also shown in Figure 12.

#### 4.2.2. Change of Feature Enhancement Mechanism

**Figure 13:**
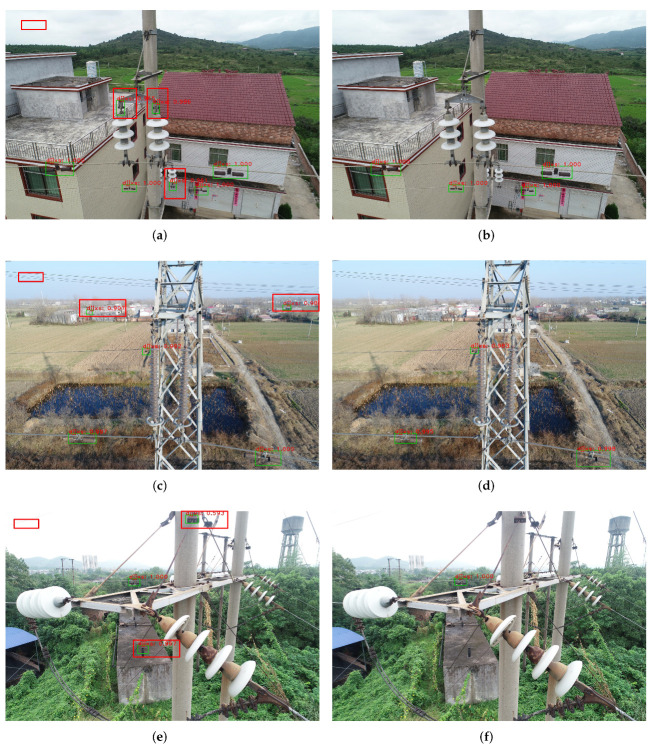
Comparative results for evaluating the feature enhancement mechanism, where (**a**,**c**,**e**) are the results without the mechanism, (**b**,**d**,**f**) are the results of using the mechanism.

**Figure 14:**
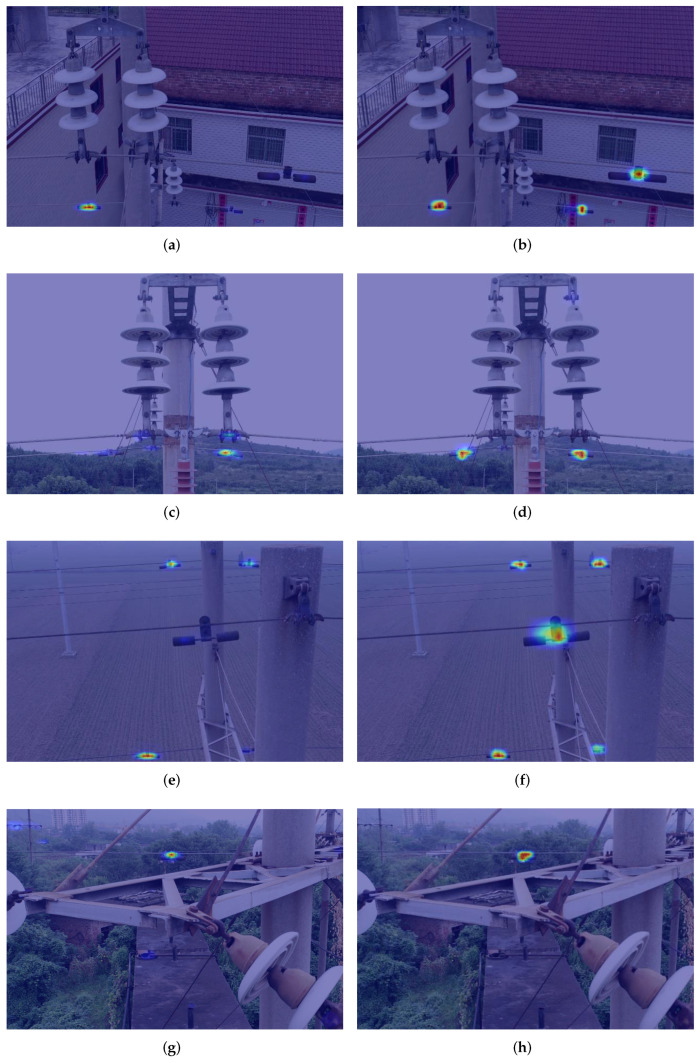
Feature heat maps of the results in Figure 13, where (**a**,**c**,**e**,**g**) are the results without the mechanism, (**b**,**d**,**f**,**h**) are the results of using the mechanism.

Figure 13 clearly shows the detection results adding the feature enhancement mechanism. The corresponding feature heat maps are shown in Figure 14. The results demonstrate that the false detection in the red box can be better improved by employing the feature enhancement mechanism. Obviously, the feature enhancement mechanism can fully utilize the correlation of region proposals to enhance the feature information of the target region. Meanwhile, it improves the ability of accurate recognition of the target objects, which can well solve the problem of false detection in complex background environments.

### 4.3. Comparative Results

**Figure 15:**
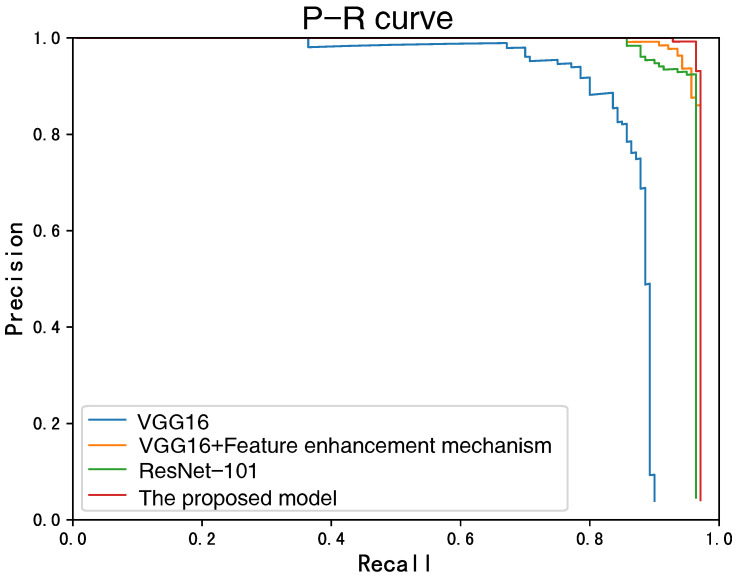
P-R curve of the models for comparison. The closer the curve is to the right-hand top corner, the better the detection performance will be.

We also employ the two indexes, Recall and Precision, to numerically evaluate the detection performance, as listed in Table 2. To provide a straightforward comparison, we further plot the P-R curve of the methods, as shown in Figure 15. The formulations of the two indexes are as follows:

(7) $$Recall=\frac{TP}{TP+FN}$$

(8) $$Precision=\frac{TP}{TP+FP}$$

where *TP* (True Positive) represents positive samples that are correctly classified; *FP* (False Positive) is negative samples wrongly categorized as positive ones; *TN* (True Negative) is the negative examples that are correctly classified; *FN* (False Negative) represents negative samples wrongly categorized as positive ones.

From Table 2 and Figure 15, the detection accuracy of the proposed model is much higher than the other methods. This further demonstrates that the proposed model can effectively improve the feature representation and enhance the feature information of the region of interest as well. Consequently, a more accurate and reliable detection of rusted fittings can be achieved.

**Figure 16:**
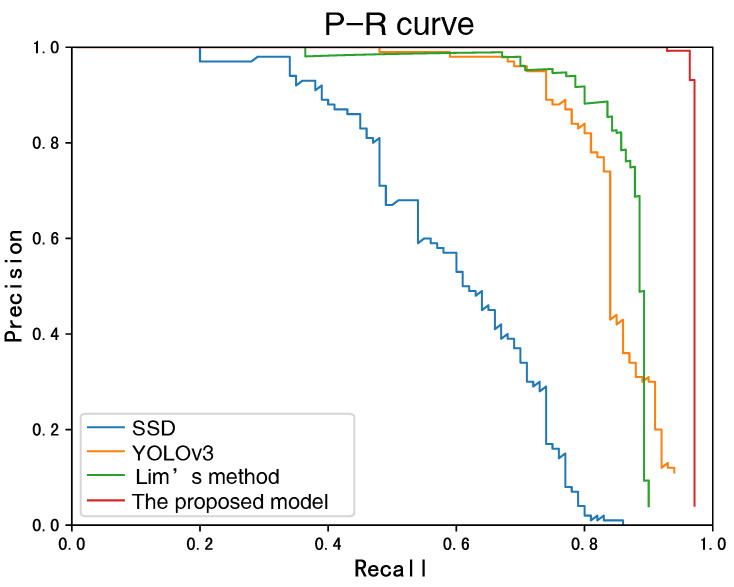
P-R curve of the four object detection methods.

Furthermore, we compare the proposed model with two typical object detection algorithms SSD and YOLOv3. We also introduce a state-of-the-art small-size object objection algorithm, called Lim’s method [12], for comparison. The detection results of the four methods are shown in Figure 16. No surprisingly, the proposed model gets the best detection performance, which proves again the effectiveness of the feature enhancement mechanism.

## 5. Conclusions

In this paper, a new robust Faster R-CNN model is proposed for the rust detection of transmission line fitting. This model aims at solving the two challenges of the rust detection: disturbance of complex environment and small size of fitting object. The proposed model focuses on the feature enhancement based on the obtained region proposals. With the proposed feature enhancement mechanism, the feature representation of the rusted fittings can be improved in an targeted mode. Moreover, the mechanism is of good application universality, since it can work on different kinds of two-stage detection architectures. With self-learning the rich information about the fitting object, the detection robustness as well as accuracy can then be developed with much lower missing detection rate and false detection rate. Then the reliability of the detection results can be much improved. The proposed model is easy to implement and has better deployment capacity for real-world applications, especially for online scenarios.

In future works, we plan to exploit the structured information about fittings. It can be observed that the appearance of fittings must be accompanied with transmission lines, which indicates sort of structured information. This information is believed beneficial for the rust detection. Moreover, for an actual engineering, the trustworthy decision is more preferable. Interpretability analysis will be applied to the rust detection. How to understand the detection results is another interesting problem. Online rust detection should be also paid more attention since online tour-inspection is an actual demand for UAV applications. In our current engineering, the online detection task is made by loading the offline-trained detection algorithm into the UAV, which motivated our study in this paper, i.e., enhancing the robustness of Faster R-CNN. We think another feasible solution is updating the detection algorithm online with the sequentially-collected images, i.e., in an incremental mode. For example, if the UAV tours around some special terrains, such as forest, villages, rivers, etc., the images with such terrain characteristics should bring more kinds of feature representation for the detection algorithm. The detection model is then required to be updated automatically and incrementally. How to incrementally update the online detection model is interesting, of course, not easy to realize, for the online tour-inspection. We will study this problem in the future work.

## Figures and Tables

**Table 1:** Structure of the ResNet-101 network used in this paper.

Feature Layer	Size of Feature	ResNet-101
Conv1	112×112	7×7,64, stride 2
Conv2_x	56×56	3×3maxpooling,stride2
		$\left[\begin{array}{cc} \begin{array}{c} 1\times 1\\ 3\times 3\\ 1\times 1 \end{array} & \begin{array}{c} 64\\ 64\\ 256 \end{array} \end{array}\right]\times 3$
Conv3_x	28×28	$\left[\begin{array}{cc} \begin{array}{c} 1\times 1\\ 3\times 3\\ 1\times 1 \end{array} & \begin{array}{c} 128\\ 128\\ 512 \end{array} \end{array}\right]\times 4$
Conv4_x	14×14	$\left[\begin{array}{cc} \begin{array}{c} 1\times 1\\ 3\times 3\\ 1\times 1 \end{array} & \begin{array}{c} 256\\ 128\\ 512 \end{array} \end{array}\right]\times 23$
Conv5_x	7×7	$\left[\begin{array}{cc} \begin{array}{c} 1\times 1\\ 3\times 3\\ 1\times 1 \end{array} & \begin{array}{c} 512\\ 512\\ 2048 \end{array} \end{array}\right]\times 3$
	1×1	Average pooling, 1000-d FC, Softmax

**Table 2:** Numerical comparison of the Faster R-CNN model based on the backbone network.

Model	Precision (%)	Recall (%)	TP	FP	FN
Faster R-CNN with VGG16	86.09%	77.28%	414	102	122
Faster R-CNN with ResNet-101	95.88%	90.91%	955	91	95
Faster R-CNN with VGG16 and Feature enhancement mechanism	96.75%	94.23%	1190	109	72
The proposed model	97.07%	96.61%	1390	103	48

## Data Availability

Not applicable.
